# Unlocking Colorectal Cancer Diagnostics With Circulating Progastrin (hPG80): A Scoping Review

**DOI:** 10.1177/11772719261478092

**Published:** 2026-09-25

**Authors:** Valentin Butnari, Deepak Kumar Dinkar, Jayan Dewantha Jayasinghe, Manthan Patel, Nirooshun Rajendran, Sherif Raouf, Madapura Marulasiddappa Pradeepa, Mohamed Adhnan Thaha

**Affiliations:** 1 The Centre for Neuroscience, Surgery, and Trauma, Faculty of Medicine and Dentistry, Blizard Institute, National Bowel Research Centre, 536118 Queen Mary University of London, England, United Kingdom of Great Britain and Northern Ireland; 2 Department of Colorectal Surgery, The Royal London Hospital, 9744 Barts Health NHS Trust, London, England, United Kingdom of Great Britain and Northern Ireland; 3 The Centre for Genomics and Child Health, Blizard Institute, Faculty of Medicine and Dentistry, 536118 Queen Mary University of London, England, United Kingdom of Great Britain and Northern Ireland; 4 Department of Surgery, 6965 Barking Havering and Redbridge University Hospitals NHS Trust, Rom Valley Way, Romford, London, England, United Kingdom of Great Britain and Northern Ireland; 5 Department of Oncology, St. Bartholomew’s Hospital, 9744 Barts Health NHS Trust, West Smithfield, London, England, United Kingdom of Great Britain and Northern Ireland

**Keywords:** hPG80, progastrin, cancer, blood biomarker, diagnosis, colorectal cancer, liquid biopsy

## Abstract

Colorectal cancer (CRC) represents a significant global health concern, ranking as the third most diagnosed cancer and the second leading cause of cancer deaths in the UK. Although population-based bowel cancer screening programs (BCSP) facilitate early diagnosis, uptake remains variable. Blood-based biomarkers, such as human progastrin 80 (hPG80), offer a patient-friendly approach to enhance screening compliance and clinical management. This scoping review, conducted according to PRISMA-ScR guidelines and registered in PROSPERO (CRD420250654595), searched Ovid Medline/PubMed, Ovid Embase, and Cochrane databases from January 1990 to January 2025. Studies assessing plasma hPG80 as a CRC biomarker were included, while reviews, in vitro studies, and animal models were excluded. Quality assessment was performed using the Newcastle-Ottawa Scale, and findings were synthesized narratively. Five articles encompassing 2,692 participants met the inclusion criteria. The included studies consistently demonstrated significant overexpression of hPG80 in CRC patients compared to non-cancer controls, with an increased median progastrin concentration in the CRC group (27.5 pmol/L) compared to patients with polyps or normal controls. Furthermore, hPG80 levels decreased significantly post-surgery (from 272.3 ± 12.0 pmol/L to 161.3 ± 11.0 pmol/L), reflecting its sensitivity to tumor burden and potential for treatment monitoring. Methodologically, literature has shifted from early radioimmunoassays to safer, cost-effective enzyme-linked immunosorbent assays (ELISA). A meta-analysis was not feasible due to variations in analytes, assays, populations, thresholds, and a lack of CRC-specific sensitivity, specificity, or area under the curve data. This review highlights hPG80’s promise as a reliable, reproducible blood-based biomarker for CRC integration into BCSPs and symptomatic pathways. However, optimal thresholds, stage-specific correlation, molecular subtypes, and cost-effectiveness remain unanswered. The ongoing MOTION trial (NCT07035691) is currently evaluating hPG80’s performance within UK symptomatic suspected colorectal cancer referral pathways.

## 1. Introduction

Colorectal cancer (CRC) is a serious global health concern. According to the latest data reported by the Global Cancer Observatory, it is the third most diagnosed cancer and the second leading cause of cancer-related deaths worldwide.^1,2^ Within the United Kingdom, nearly 44,100 new cases are diagnosed annually, translating to 120 cases per day.^3^ Current predictions made by the World Health Organisation state that this burden will increase, potentially reaching more than 47,700 diagnoses per year by 2040.^4,5^ While the current incidence rates in the UK are reported to be the highest in the 80 years plus age group, emerging research indicates a concerning trend (1 in every 20 new cases) of CRC increasingly affecting younger individuals, with a growing number of cases reported below the age of 50.^3,6^

CRC typically develops due to a combination of genetic and environmental influences, with several factors increasing its risk. Some risk factors are modifiable, including diet, obesity, lack of physical activity, smoking, and alcohol use. Other non-modifiable risk factors include sex, ethnicity, a history of inflammatory bowel disease, a personal or family history of polyps or cancer, inherited conditions such as Lynch syndrome, and genes involved in cancer development.^7,8^ CRC often develops from pre-existing colorectal adenomas (CAAs, also known as polyps) through a progression known as the adenoma-carcinoma sequence.^9^ This sequence involves gradual accumulation of genetic changes that transform normal colon tissue into invasive cancer, a multi-step process which often takes many years (Figure 1). Many deaths from CRC can be prevented by the early detection and treatment of CAAs or early-stage cancers before they progress to more advanced disease.^10,11^

**Figure 1.**
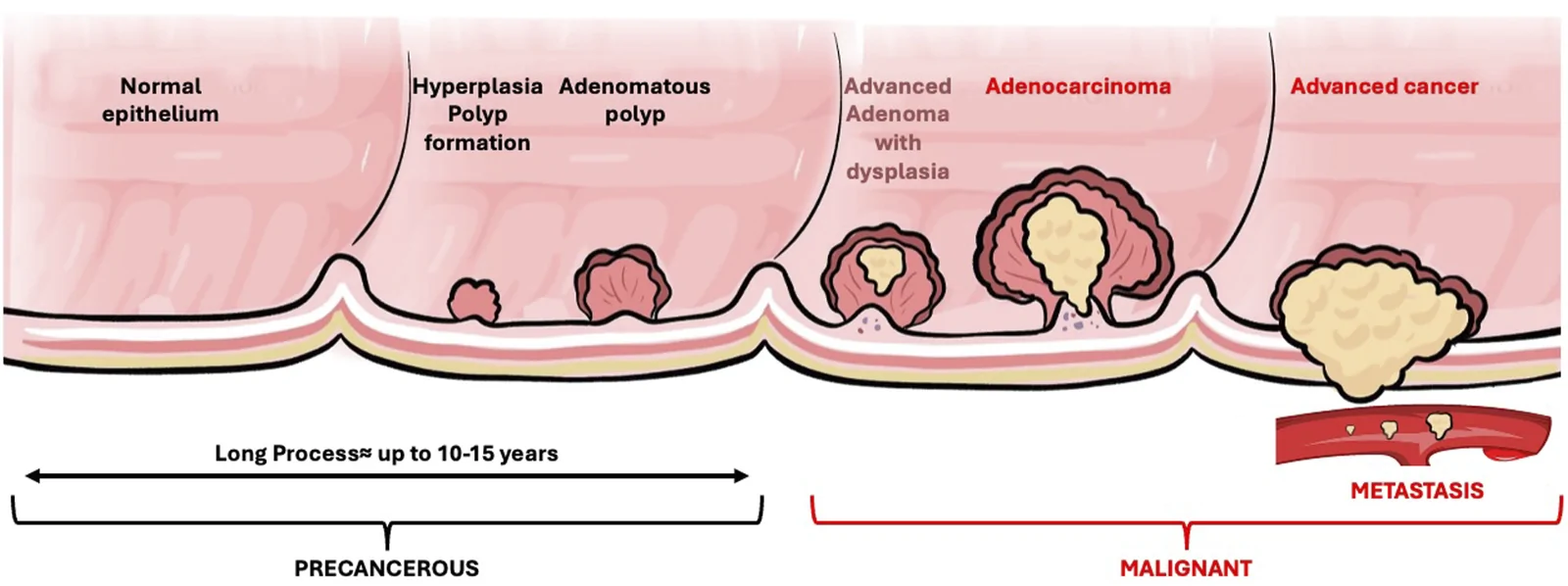
Colorectal cancer progression

The early detection of CRC relies on two key strategies, early diagnosis in symptomatic patients and population-based screening.^12-14^ Currently, a stool test is commonly used as an initial triage tool, and colonoscopy is considered as the gold standard investigation. Other methods employed include a virtual colonoscopy. Less invasive tests such as blood-based biomarkers are emerging concepts that are under investigation (Figure 2).

**Figure 2.**
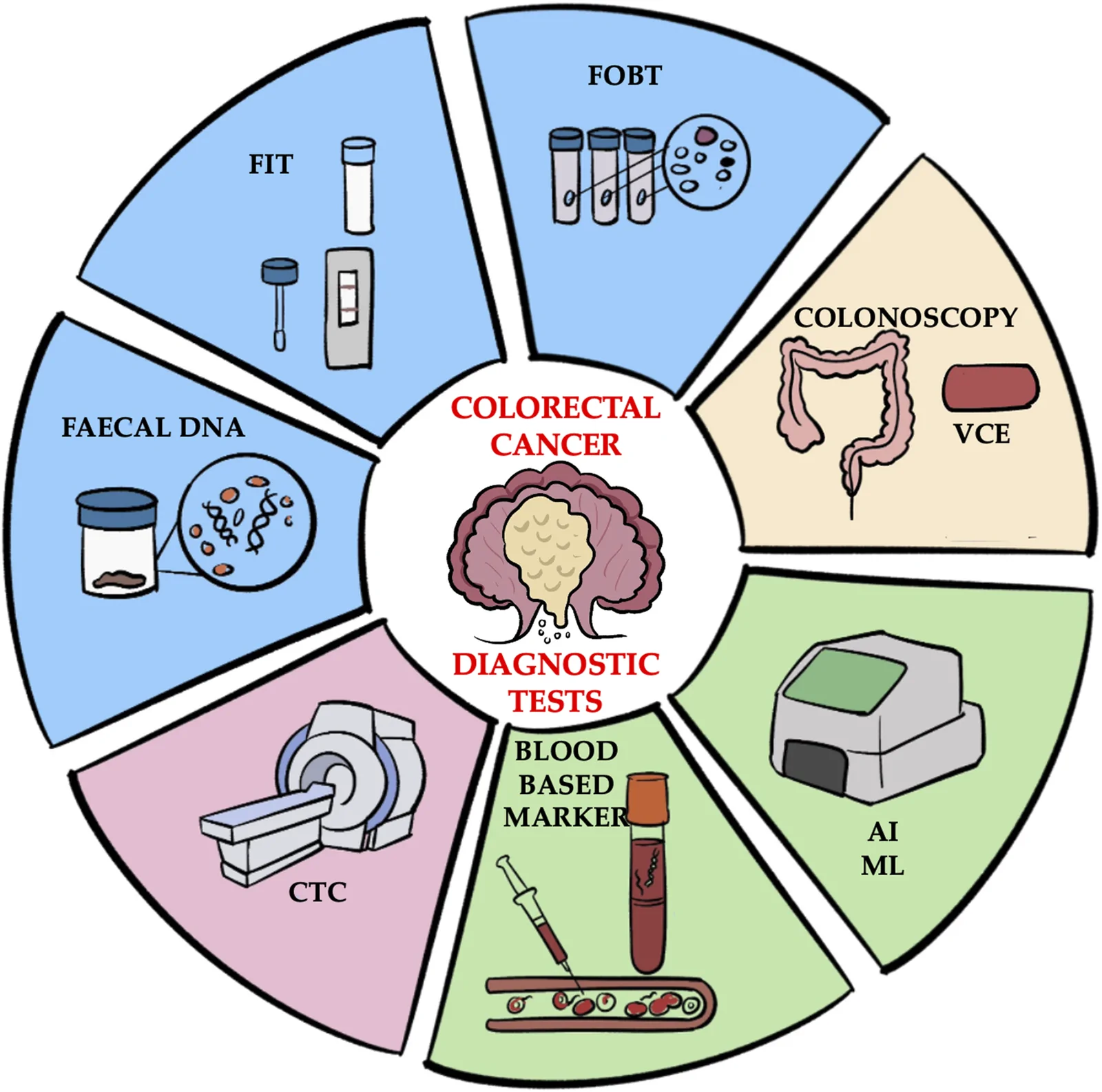
Colorectal cancer diagnostic and screening modalities

The guidelines for CRC screening differ worldwide with variations in population-specific risk profiles, availability of healthcare resources, and patient and society values.^15^ In the majority of countries around the world, including the UK, the faecal immunochemical test (FIT) is the most widely adopted first test for systematic CRC screening.^16-18^ However, there is uncertainty regarding the optimal FIT threshold within different clinical pathways, which highlights the potential for future research. On the other hand, colonoscopy is the most common screening modality in the United States of America as well as other countries like Germany and Poland where opportunistic screening is employed. Despite its high sensitivity and specificity for CRC detection and polyp removal, colonoscopy faces limitations due to its high associated costs, invasive nature requiring bowel preparation, and high healthcare resource demand, impacting the overall acceptability of this screening method.^19^ The UK National Health Service (NHS) Bowel Cancer Screening Programme (BCSP) reported an uptake of 67.6% in 2023-24, with coverage reaching 71.8% at the end of March 2023.^20^ Despite evidence showing that screening significantly reduces mortality, CRC screening rates are currently lower than the desired 80% across all populations.^21^ Hence, there is still a critical unmet need for multimodal CRC screening strategies capable of enhancing current screening participation rates worldwide.

Recent evidence from randomized control trials suggests that blood-based testing holds promise in improving adherence to CRC screening programs. Consequently, CRC research is increasingly focused on utilizing individualized genetic risk profiles with the aim of enhancing the diagnosis and prognosis of this disease.^22-24^ Numerous studies have investigated molecular markers that may contribute to the development of personalized medicine to improve disease prediction and patient outcomes.^25-27^ Liang et al suggested in their randomised control trial that patients prefer blood tests for CRC screening over other methods such as FIT or colonoscopy.^28^

The rising incidence of early-onset colorectal cancer, defined as disease diagnosed before the age of 50, has sharpened the need for non-invasive detection. This trend falls largely outside the age range of most population screening programmes, so many cases present symptomatically and at a later stage. The Delphi Initiative for Early-Onset Colorectal Cancer (DIRECt) produced the first consensus recommendations for this group and called for prompt risk stratification and timely symptom workup in adults younger than 50.^29^ Blood-based detection is one route to that goal. Several cell-free DNA platforms have now been validated in large prospective cohorts. The Shield assay reported a sensitivity of 83 percent for colorectal cancer with a specificity of 90 percent in the ECLIPSE study and became the first blood test approved by the United States Food and Drug Administration for primary colorectal cancer screening.^30^ A multiomic cell-free DNA test reported a sensitivity of 79 percent for colorectal cancer with a specificity of 92 percent for non-advanced neoplasia in the PREEMPT CRC study.^31^ These platforms share a common limitation, namely low sensitivity for advanced precancerous lesions, and they detect tumour-derived nucleic acids rather than a secreted protein. hPG80 is mechanistically distinct, since it is a secreted oncogenic peptide rather than circulating tumour DNA, and it is measured by a simple enzyme-linked immunosorbent assay. It therefore warrants evaluation as a complementary, rather than competing, candidate within this evolving landscape.

One promising plasma biomarker under investigation is hPG80, an extracellular form of progastrin.^32^ Before 2020, the medical literature referred to progastrin without explicitly differentiating between its intracellular role in digestion and extracellular detection within the context of malignancy.^33^ The association between progastrin and cancer was first reported by Bardram in 1990, who investigated circulating progastrin levels in patients with Zollinger-Ellison syndrome.^34^ His findings indicated that elevated progastrin levels could serve as an early indicator of malignancy, suggesting that total progastrin levels more accurately reflect tumour gastrin synthesis than conventional measurements of alpha-amidated gastrin.^34^ Since then, very few studies have investigated this marker.^35-38^ To date, the largest multicentre study on hPG80 was conducted by You et al.^33^ Their group examined samples from 1546 non-fasting patients with 11 different cancer types. They suggested that hPG80 release from tumour cells is crucial for its oncogenic activity, which subsequently reinforces the hypothesis that progastrin has a substantial impact, particularly on the disruption of cell junctions, cell proliferation, inhibition of apoptosis, regulation of cancer stem cells, and angiogenesis, leading to tumour development and progression.^33,39-43^ This group stated that hPG80 has functions beyond digestion that are distinct from progastrin’s role as an intracellular precursor of gastrin. The name of hPG80 brings clarity for the distinction between the human progastrin intracellular digestive role and its extracellular form present in blood stream of cancer patients which despite the name is no longer gastrin precursor. ‘h’ signifies human origin, ‘PG’ indicates the name progastrin and ‘80’ indicates its 80 amino acid length. This nomenclature is proposed by You et al.^33^ Siddheshwar et al first identified and quantified progastrin in the blood of CRC patients in 2001.^35^ This was followed by a study by Konturek et al in 2002, who measured progastrin levels in patients with CRC before and after surgery.^36^ Their findings revealed elevated progastrin levels preoperatively compared to the controls, with a return to normal levels postoperatively. Paterson et al then reinforced the hypothesis that concentrations of gastrin precursors are increased in patient with high risk of developing CRC.^38^ In this manuscript, we aim to describe and present the available literature on the diagnostic potential of plasma or serum-based progastrin (hPG80) in the diagnosis of CRC and present the design of a new trial to test the hypothesis of hPG80 as blood biomarker within a clinical pathway.

## 2. Materials and Methods

### 2.1. Study Selection

An electronic search of the Ovid Medline/PubMed, Ovid Embase, EMBASE, and Cochrane databases was performed to identify current literature available on circulating progastrin levels in patients with CRC. We used a search strategy combining medical subject heading terms with Boolean operators (AND, OR, and NOT). The full electronic search strategy for each database, with field codes and the number of records returned, is reported in Supplementary Appendix 1 and identified all relevant articles published between January 1990 and January 2025 (Appendix 1). Duplicates were then removed. Based on the title and abstract, manuscripts of interest were obtained for a full-text review. Only the full-text manuscripts were included. Additional articles were identified by manually searching the references of original and review articles.

### 2.2. Protocol and Registration

Our systematic scoping review followed the Preferred Reporting Items for Systematic Reviews and Meta-Analyses extension for Scoping Reviews (PRISMA-ScR) guidelines.^44^ The review protocol was registered in the International Prospective Register of Systematic Reviews (PROSPERO) under number CRD420250654595.

### 2.3. Inclusion and Exclusion Criteria

Original studies that assessed plasma levels of gastrin or hPG80 as biomarkers in individuals with CRC, colorectal polyps, or non-cancerous conditions were included. We excluded studies that did not explore gastrin or hPG80 as biomarkers for the diagnosis of CRC, review articles, *in-vitro* studies, animal model studies and studies focusing on non-blood biomarkers.

### 2.4. Outcomes

The primary outcome measure of this study was to evaluate the diagnostic potential of plasma levels of gastrin or hPG80 as biomarkers in differentiating individuals with CRC or colorectal polyps from non-cancerous controls.

The secondary outcomes are:

1. Investigating the relationship between hPG80 level and the stage of CRC.
2. Assessment of the ability of the hPG80 blood test to differentiate high-risk from low-risk polyps
3. Describe a threshold value for the hPG80 blood test for CRC diagnostic purposes.
4. Evaluating the reproducibility of the hPG80 blood test.
5. Assessing the clinical utility and limitations of the hPG80 blood test.

### 2.5. Data Extraction

Title and abstract screening, full-text screening, and data extraction were each performed in duplicate and independently by two reviewers (VB and DKD). Each manuscript was evaluated for eligibility, and extracted data encompassed study characteristics (first author, publication year, country, and study design), patient demographics (age, gender ratio, and CRC stage), and the key findings of each article. Agreement between reviewers across the screening stages was substantial. Discrepancies were resolved through consensus and, where consensus could not be reached, by adjudication from a third reviewer (MAT); adjudication was required for three of the records taken to full-text assessment, and in those cases the final eligibility decision was reached by majority.

### 2.6. Quality Assessment

The methodological quality of the included studies was appraised using the Newcastle-Ottawa Quality Assessment Scale (NOS), a standardized tool designed to evaluate non-randomized study designs. The NOS assigns a star rating to studies based on three domains: participant selection, comparability of groups, and outcome assessment. Studies were categorized as having a low, moderate, or high risk of bias according to their total star rating: 7-9 stars, 4-6 stars, and <4 stars, respectively.^45^

We additionally applied the Quality Assessment of Diagnostic Accuracy Studies 2 (QUADAS-2) tool to all five included studies. Risk of bias was rated across the four QUADAS-2 domains (patient selection, index test, reference standard, and flow and timing), while applicability concerns were assessed across the first three domains.

### 2.7. Rationale for a Scoping Review Without Quantitative Synthesis

We assessed whether a meta-analysis of diagnostic accuracy or of circulating analyte concentrations was feasible. It was not, for three reasons. First, the index analyte differed across studies. Siddheshwar et al, Konturek et al, and Paterson et al measured progastrin by radioimmunoassay using antibodies that were not counter-selected against gastrin, reporting several distinct peptides (progastrin, amidated gastrin, glycine-extended gastrin, and total gastrin). Conversely, You et al and Chauhan et al measured hPG80 by a sandwich enzyme-linked immunosorbent assay (DxPG80) using antibodies selected to bind progastrin alone. Pooling concentrations measured by non-equivalent assays against different molecular targets would not produce an interpretable summary estimate. Second, no two studies reported the same diagnostic accuracy metric in a colorectal cancer population. None of the five studies reported a colorectal cancer-specific sensitivity, specificity, or area under the curve. The accuracy figures in You et al were derived from a pooled pan-cancer cohort and were not broken down for the colorectal subgroup, whereas the accuracy figures in Chauhan et al described neuroendocrine neoplasms. Because a two-by-two contingency table for colorectal cancer detection could not be reconstructed for any study, a bivariate or hierarchical summary receiver operating characteristic model could not be fitted. Third, the populations, comparators, and thresholds varied widely. Comparators ranged from endoscopically normal controls and benign polyp cases to hereditary syndrome carriers and healthy blood donors of differing ages. The reported thresholds ranging from single-digit picomolar concentrations to 15 pmol/L and 154 pmol/L reflected these incompatible designs and assays rather than a transferable cut-off. Forcing a pooled estimate across these disparate sources would introduce severe clinical, methodological, and analytical heterogeneity, generating a spuriously precise figure. A scoping review that maps the evidence and explains its limits was therefore the appropriate design, consistent with the registered protocol.

## 3. Results

### 3.1. Study Characteristics

Our literature search identified 55 relevant articles. Following a duplication check and preliminary screening of article titles and abstracts, a total of five articles describing circulating progastrin in patients with CRC and colorectal polyps were retrieved and fully evaluated (Figure 3). These five studies included 2692 participants, with sample sizes specific for CRC ranging from 5 to 305 individuals (Table 1).

**Figure 3.**
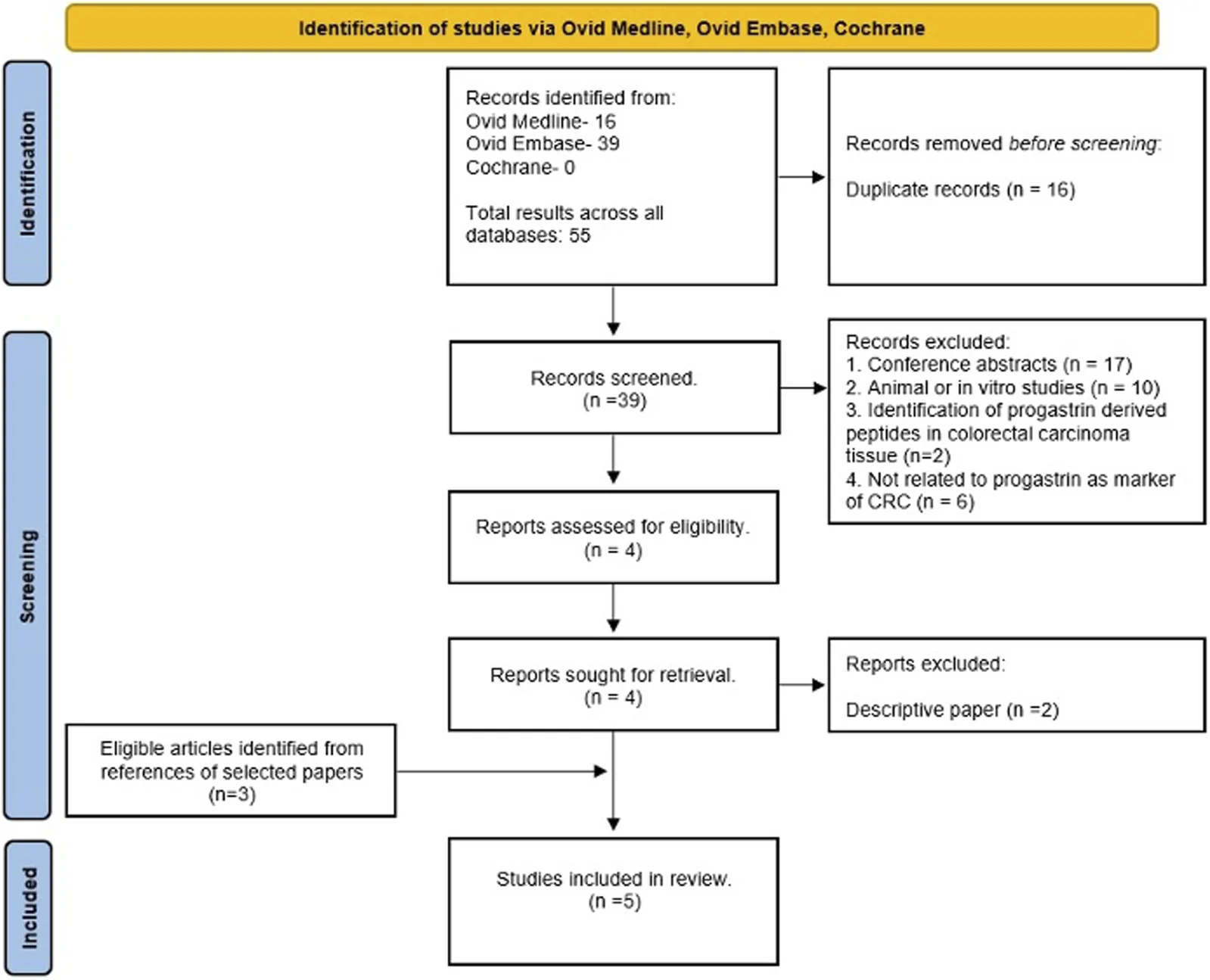
PRISMA flow diagram

**Table 1.** Characteristics of included studies

First author	Year	Country	Study design	Sample type (s)	Total no.	CRC (no. of patients)	Non-CRC (no. of patients)		Age (median ± s.d./(range), years	Gender male: female	CRC stage (no. of patients)	Key findings
							Polyps	Controls				
Siddheshwar et al.^35^	2001	United Kingdom	Prospective diagnostic study. Comparing plasma progastrin, amidated gastrin, or glycine extended gastrin in patients with colorectal cancer or colorectal polyps or controls	Plasma	139	57	Total 29 Type of polyp: 1. Tubulovillous adenoma-24 2. Tubular adenoma-11 Degree of dysplasia Mild-10 Moderate-22 Severe-3	53 (10 suffered from diverticular disease)	CRC 70 (42-84) Polyps 65 (42-86) Control 56 (27-79)	32:25 19:10 21:32	Dukes classification A-7 B-21 C-27 and declared inoperable- 2	Plasma levels of progastrin, but not amidated gastrin or glycine extended gastrin, are significantly elevated in patients with colorectal cancer compared with those with colorectal polyps or controls, irrespective of their H pylori status. Measuring plasma progastrin levels in patients with colorectal cancer is warranted
Konturek et al.^36^	2002	Poland	Observational treatment response study determining plasma levels of progastrin and amidated gastrin in CRC patients before and after tumour removal; examine tumour concentrations and gene expression	Plasma and Tissue	150	50	-----	100	CRC 65 (43-70) Control 62 (44-70)	28:22 57:43	Dukes classification A-5 B-23 C-22	The CRC and its margin contain large amounts of progastrin and show gene expression of gastrin, CCK_B_−R mRNA, and COX-2 mRNA. Removal of the CRC markedly reduces the plasma concentrations of progastrin. H Pylori infection rate is higher in CRC and may contribute to colorectal carcinogenesis *via* enhancement of progastrin and gastrin release. Plasma progastrin concentrations might serve as a biomarker of CRC
Paterson et al.^38^	2014	Australia	Observational diagnostic study investigated circulating gastrin concentrations in patients at increased risk of developing CRC. Patients grouped into familial adenomatous polyposis, hereditary non-polyposis colorectal cancer, cluster of common colorectal cancers, personal history and/or family history of adenomatous polyps or CRC, and controls	Plasma	278	5	Familial adenomatous polyposis (n=20), hereditary non-polyposis colorectal cancer (n=53). Patients from all groups presenting with an adenomatous polyp also mentioned	Healthy controls (n=42). Patients from all groups presenting with an adenomatous polyp also mentioned.	FAP 35.6 ± 2.6 HNPCC 48.0 ± 1.7 CCC 54.3 ± 3.4 PH/FH 55.7 ± 1.2 Controls 44.6 ± 2.3 Subjects without polyps/CRC 51.1 (49.0–53.2) Subjects with polyps/CRC 55.5 (51.1–59.8)	FAP 11:9 HNPCC 23:30 CCC 5:8 PH/FH 70:80 Controls 20:22	-----	Concentrations of gastrin precursors are increased in particular groups with an increased risk of developing CRC. FAP patients had significantly higher median values of total gastrin and gastrin-amide compared to controls. Patients with personal/family history of adenomatous polyps or CRC had higher circulating total gastrin compared to controls. Patients with an adenomatous polyp had higher concentrations of total gastrin, progastrin, and gastrin-amide than patients without polyps. Patients exhibit an elevated proportion of Gly gastrin amide that implies a reduction in the extent of amidation in this group.
You et al.^33^	2020	France	Observational diagnostic study. Investigated hPG80 expression in various tumour types, cancer stem cells self-renewal, and hPG80 detection in blood samples from patients with 11 different tumour types	Plasma and Tissue	1546	305 with CRC out of 1546 cancer cases included in the study	-----	557 total controls samples	CRC 64 (23-93) Control 35 (18-70)	174:123 310:247	American Joint Committee on Cancer Classification I-25 II-73 III-27 IV- 169 Undetermined stage- 11	The oncogenic and druggable hPG80 (circulating progastrin) is overexpressed in multiple cancers and detected in the blood of patients. hPG80 was present in 11 tumour types tested. hPG80 neutralization decreased CSC self-renewal. hPG80 was detected in the blood of patients at significantly higher concentration than in healthy blood donors. Strong association between longitudinal hPG80 concentration changes and anti-cancer treatment efficacy. hPG80 is a promising cancer target for therapy and disease monitoring. This study suggests that anti hPG80 neutralising antibodies decreases in vitro cell proliferation and sphere formation of tumour cell lines. This confirms that hPG80 is indeed released from tumour cells.
Chauhan et al^37^	2022	USA	Observational diagnostic study. Examined hPG80 as a novel blood-based biomarker for detection of poorly differentiated neuroendocrine carcinoma (NEN) and well differentiated neuroendocrine tumours. Quantified plasma hPG80 from 95 stage IV NEN patients and compared with two cohorts of healthy donor controls	Plasma	579	92 Neuroendocrine carcinoma as within GI tract was not specified CRC precisely	-----	Healthy donor controls aged 50-80 (n=252) and 18-25 (n=137). Median hPG80 in NENs patients were 5.54 PM compared to 1.5 PM for the 50-80 years controls	GI NETs 62 (37-86) Control 38 (18 -80)	n/a 178:211	-----	hPG80 is a novel blood-based biomarker for NENs. Increased levels of hPG80 in all sub-types of NENs, with high sensitivity and specificity demonstrated. Plasma hPG80 in NENs may be a diagnostic blood biomarker for both low- and high-grade NENs

The studies were conducted across multiple countries including France, Poland, the United States of America, the United Kingdom, and Australia. The populations primarily consisted of patients diagnosed with CRC and polyposis syndromes analysed as a subgroup in two studies, with one study including patients with benign polyps for comparison. Key demographic and clinical characteristics, such as CRC stage, tumour location, and presence of metastasis were reported in varied details by most of the studies with most including adults over 30 years. For instance, You et al.^33^ included a large cohort of 305 CRC patients with detailed staging information, whereas Siddheshwar et al.^35^ focused on a smaller group of 57 patients, primarily examining plasma progastrin levels.

### 3.2. Quality Assessment of Included Studies

The methodological quality of the included studies was assessed using the Newcastle-Ottawa Scale (NOS) (Table 2). Overall, three studies were rated as high quality and two were rated as moderate quality, indicating a generally robust body of evidence. However, while assessing the bias using the QUADAS-2 appraisal (Supplementary Table 1) this revealed that all five studies carried a high or unclear risk of bias in the patient selection domain. This was because every study used a case-control design that enrolled patients with confirmed cancer separately from controls, a methodology known to inflate diagnostic accuracy. While the reference standard domain was at low risk of bias in studies using histological confirmation, issues regarding flow and timing were frequently unclear. These ratings reinforce that the present evidence base cannot support a pooled estimate of diagnostic accuracy.

**Table 2.** Newcastle-Ottawa quality assessment

Study	Selection	Comparability	Outcome	Scoring
Siddheshwar et al.^35^	4	2	2	8
Koturek et al.^36^	3	2	2	7
Peterson et al.^38^	4	2	2	8
You et al.^33^	4	2	2	8
Chauhan et al^37^	3	1	2	6

### 3.3. Progastrin Expression in CRC Diagnosis and Prognosis

All five studies demonstrated that circulating progastrin (hPG80) is significantly overexpressed in patients with CRC compared to healthy controls or individuals with benign polyps. Siddheshwar et al.^35^ reported that progastrin levels were significantly elevated in CRC patients (median: 27.5 pmol/L) compared to both the polyp (<15 pmol/L) and control (<15 pmol/L) groups (p = 0.0001). Importantly, this study found that progastrin levels were elevated in CRC patients irrespective of their Helicobacter pylori (Hp) status, suggesting that Hp infection is not the primary cause of hyperprogastrinemia in these patients. Additionally, Siddheshwar et al.^35^ observed that the median plasma circulating progastrin levels were significantly higher in patients with moderately dysplastic polyps (38 pmol/L) than in those with mildly (15 pmol/L) or severely (15 pmol/L) (p = 0.05) dysplastic polyps indicating a potential role in differentiating polyp risk levels.

Konturek et al.^36^ further supported these findings, showing that progastrin concentrations in CRC patients averaged 272.3 ± 12.0 pmol/L before surgery and significantly decreased to 161.3 ± 11.0 pmol/L after tumour removal (p < 0.05). The study also reported that 86% of CRC patients had Progastrin concentrations above the cutoff point of 154.0 pmol/L before surgery, while all values fell below this threshold post-surgery (OR = 40.82; 95% CI: 2.21-75.19). This reduction in circulating progastrin levels after tumour removal suggests that CRC cells are a major source of gastrin. Konturek et al.^36^ also found that progastrin content was significantly higher in tumour tissue (34.1 ± 6.5 pmol/g) and tumour margins (46.3 ± 8.7 pmol/g) compared to intact colonic mucosa (0.5 ± 0.14 pmol/g), further supporting its role as a tumour-specific biomarker. Paterson et al.^38^ demonstrated increased circulating gastrin precursor concentrations in individuals at a higher risk of developing CRC. This included elevated total gastrin levels in patients with familial adenomatous polyposis (FAP) and those with a personal or family history of adenomatous polyps or CRC. Furthermore, patients presenting with an adenomatous polyp had higher concentrations of total gastrin, progastrin, and gastrin-amide than those without polyps, indicating its relevance in early detection or risk stratification. You et al.^33^ provided additional insights into the diagnostic utility of hPG80, reporting that CRC patients had median hPG80 concentrations of 4.36 pmol/L compared to 1.05 pmol/L in healthy donors (p < 0.0001). The study also highlighted the high sensitivity and specificity of the hPG80 assay, with a limit of detection (LoD) of 1.2 pmol/L and a limit of quantitation (LoQ) of 2.3 pmol/L. The inter- and intra-assay coefficients of variation (CV%) were below 10%, demonstrating the reliability and reproducibility of the assay.

The primary difference between the three older and two recent publications lies in the assay technology used, the older studies utilized RIA with progastrin antibodies that were not counter-selected against gastrin, whereas the recent studies used the Sandwich ELISA technology featuring antibodies specifically selected to bind only progastrin.

### 3.4. Overall Relationship Between hPG80 Levels and CRC Stage

The five included articles offer limited direct information specifically detailing the relationship between hPG80 level and CRC stage. We did not observe significant differences between the stages of cancer types tested within the included articles. Some authors hypothesized that hPG80 is not related to the tumor burden but the biological activity of the tumor.

### 3.5. Optimal Threshold Value for hPG80 Blood Test

The optimal threshold value for the hPG80 blood test was explored by You et al.,^33^ who proposed a threshold based on receiver operating characteristic (ROC) curve analysis. They reported a median hPG80 concentration of 4.36 pmol/L in cancer patients versus 1.05 pmol/L in healthy blood donors, indicating a significant difference but not proposing an optimal threshold for clinical use. Konturek et al.^36^ also identified a cutoff point of 154.0 pmol/L for circulating progastrin concentration, with 86% of CRC patients exceeding this threshold before surgery. Chauhan et al.^37^ mentioned a median hPG80 of 5.54 pmol/L for NEN (neuroendocrine carcinoma) patients compared to 1.5 pmol/L for healthy controls (50-80 years), again highlighting differences but not providing a specific threshold. Establishing a standardized threshold for hPG80 is critical for its clinical application and future studies should aim to define and validate this value across diverse populations.

### 3.6. Reproducibility of the hPG80 Blood Test

You et al.^33^ demonstrated high reproducibility, with consistent results across multiple cohorts and low intra- and inter-assay variability (CV% < 10%). Similarly, Konturek et al.^36^ reported a dramatic decrease in circulating progastrin levels after tumor removal, supporting the reliability of the assay. These findings indicate that the hPG80 blood test is a robust and reliable tool for measuring gastrin levels in different clinical settings.

### 3.7. Therapeutic Potential of hPG80

You et al highlighted the therapeutic potential of hPG80, particularly its role in cancer stem cell (CSC) regulation.^33^ This study suggested that hPG80 could be used as a target for anti-hPG80 therapies, as it has been shown to decrease CSC frequency in CRC cell lines. A monoclonal humanized therapeutic antibody targeting hPG80 is currently being developed. The study also noted that hPG80 may act synergistically with the Wnt and Ras pathways to support malignant transformation and CSC activation, further emphasizing its potential as a therapeutic target.

## 4. Discussion

Our scoping review highlights the promise of human circulating hPG80 as a novel biomarker in the early detection and clinical management of CRC. Progastrin is a precursor protein of gastrin which is encoded by the GAST gene located in 17q21 (Figure 4). Progastrin undergoes multiple maturation processes to yield a variety of peptides. The primary product of this maturation cascade is gastrin, which is predominantly synthesized by endocrine G cells located within the gastric antrum. While most gastrin production occurs in the antrum, lower levels of expression have also been observed in the duodenum.^46^ Gastrin plays a crucial role in maintaining the functional integrity of the gastric mucosa.^47,48^

**Figure 4.**
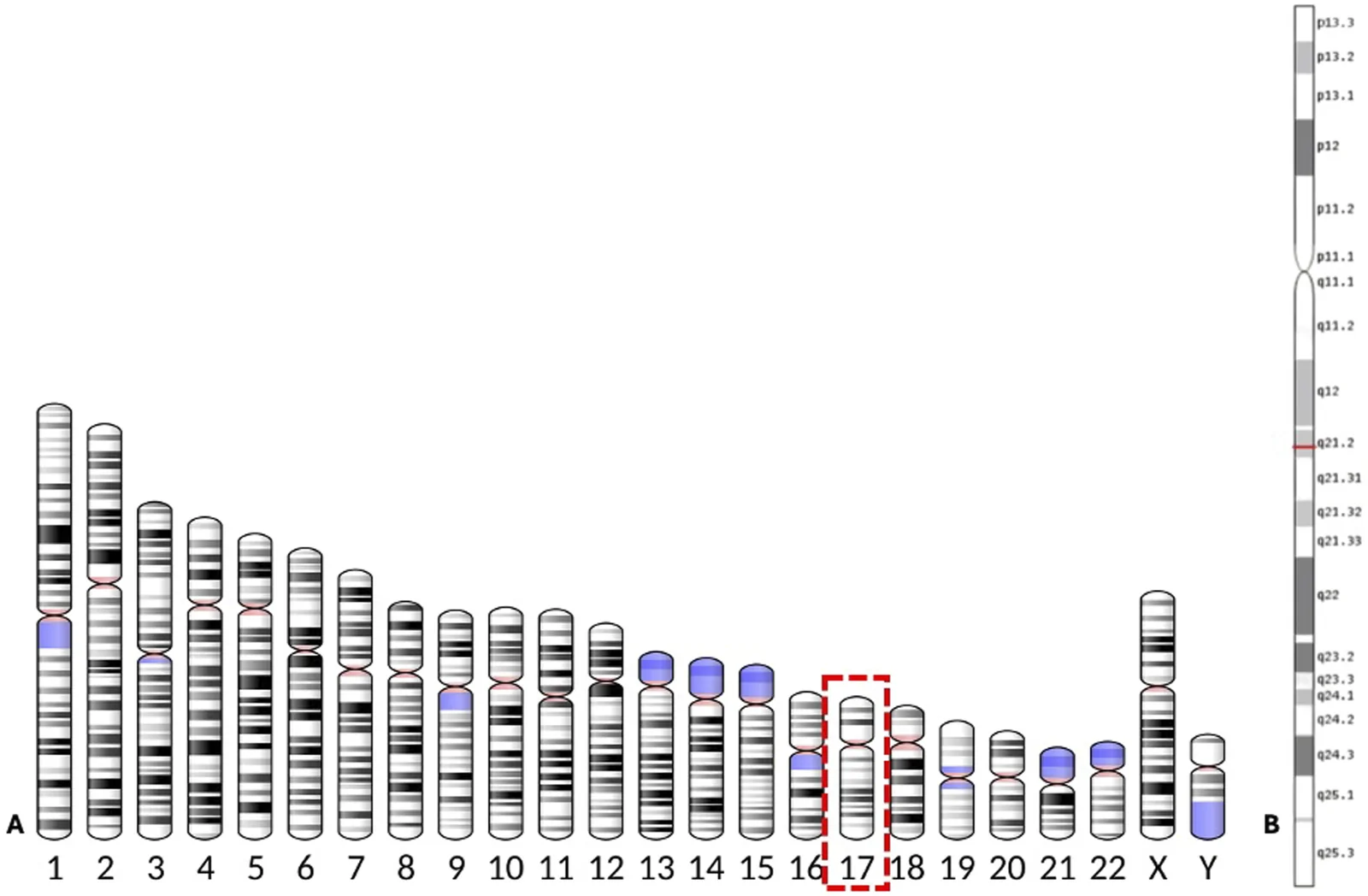
GAST gene location on human chromosome 17 A- Karyotype highlighting Chromosome 17; B-long arm locus 17q21

Under pathological conditions, the GAST gene, progastrin/hPG80, is overexpressed in human cells, resulting in hPG80 accumulation (Figure 5).^49^ hPG80 typically does not undergo proper maturation due to the absence or dysfunction of necessary enzymes.^50,51^ At the cellular level, as shown in Figure 5, preprogastrin, a 101 amino acid precursor, initiates gastrin processing in antral G cells by translocating to the trans-Golgi network following the removal of its signal sequence in the endoplasmic reticulum. Within the Golgi, progastrin undergoes modifications, such as sulfation or phosphorylation, and is then cleaved by prohormone convertase (PC), releasing the C-terminal flanking peptide (CTFP). Subsequently, carboxypeptidase E removes C-terminal arginines from the resulting immature gastrin. PC further processes glycine-extended gastrin-34 (G34-gly) into Ggly (G17-Ggly), both of which can be amidated by peptidyl-α-amidating monooxygenase (PAM), culminating in the production of amidated gastrin (G-amide), either G17 or G34.

**Figure 5.**
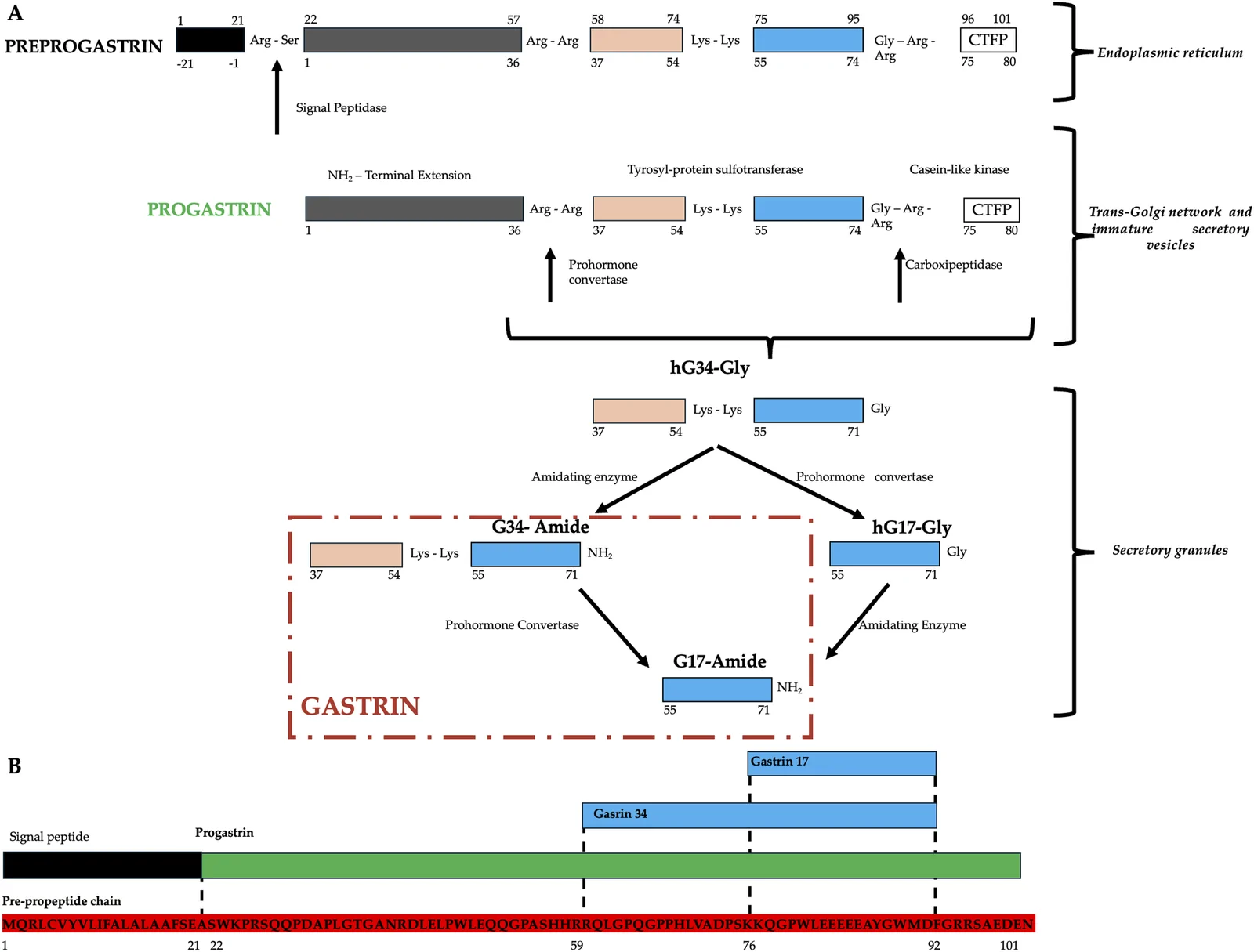
Progastrin processing A- Post-translational processing pathway of preprogastrin into progastrin and mature gastrin peptides across cellular compartments; B- Amino acid sequence mapping and domain organization of preprogastrin, progastrin, Gastrin 34, and Gastrin 17

This accumulation of unprocessed or poorly processed progastrin, potentially due to multiple genetic and epigenetic aberrations, may contribute to the development of different cancers.^48^ While typically absent in healthy individuals, an 80-amino acid progastrin variant (hPG80) is detectable in the circulation of cancer patients due to its release from tumour cells without full processing into mature gastrin.^35,52,53^ Progastrin secretion has been associated with increased cell growth, potentially promoting tumour formation.^32^ A recurrent theme identified within our scoping review describing cancer progression, particularly in the Chromosomal Instability (CIN) pathway, is the deregulation of Wnt/β-catenin pathway. Its activation, often initiated by mutations in genes such as Adenomatous Polyposis Coli can result in dysregulated cell proliferation.^49,54^ Subsequent progression often involves mutational activation of the KRAS oncogene and inactivation of the TP53 tumour suppressor gene. Notably, in CRC the Wnt/β-catenin pathway, which is aberrantly activated in 60-80% of colon cancers, directly drives the expression of the GAST gene.^50,55^

### 4.1. Potential of hPG80 in CRC Screening Programs

Integrating hPG80 into existing population-based screening initiatives, such as the NHS BCSP, presents a significant opportunity to transition from uniform screening towards a more personalized screening strategy.^56^ While the BCSP has achieved substantial public health benefits since its implementation in 2006, including reductions in CRC mortality and incidence, it remains fundamentally limited by its reliance on non-specific and non-personalized modalities like the FIT.^57^ In contrast, hPG80 may offer improved specificity for neoplastic transformation, particularly in detecting CAAs with malignant potential.

Beyond its utility in primary screening, blood-based markers such as hPG80 hold substantial promise for broader clinical application. Currently, the presence of blood in the stool typically leads to a FIT test, followed by an invasive colonoscopy in individuals with a FIT level exceeding a 10 μg/g. However, a significant proportion of these patients may have a normal colonoscopy. Combining FIT test with a blood-based marker such as hPG80 could potentially improve the sensitivity and specificity and could refine patient stratification and expedite cancer diagnosis.^58^ This is particularly relevant given the inherent limitations of colonoscopy, including its invasive nature, reliance on specialised resources, and associated waiting times. The minimally invasive nature of a blood test like hPG80 may also help mitigate the impact of socioeconomic factors, a known barrier to FIT uptake and return rates, potentially leading to more equitable screening participation.^59^ For instance, studies have shown that individuals in the most deprived quintiles are less likely to participate in CRC screening programs compared to those in the least deprived quintiles, with participation rates varying significantly across different ethnic groups. Therefore, the integration of a blood-based marker offers a multifaceted approach to improving CRC management across the continuum of care, from initial risk assessment and diagnosis to post-treatment surveillance and addressing health disparities.

### 4.2. hPG80 in Symptomatic Referral Pathways

All five studies included in our review reported significant elevations of circulating hPG80 in CRC patients compared to healthy or CAAs controls. These findings suggest that hPG80 may be sensitive enough to detect not only established malignancy but also pre-malignant lesions, such as high-grade dysplasia or advanced CAAs. This sensitivity could be leveraged within symptomatic diagnostic pathways, such as the urgent Two-Week Wait (2WW) pathway, to triage patients more effectively and reduce unnecessary colonoscopies. The potential to reduce the volume of inappropriate or negative colonoscopies is crucial, as it could conserve endoscopy resources, shorten diagnostic delays, and help healthcare systems meet referral targets (in addition to mitigating risks and low uptake associated with colonoscopy/CTC), ultimately benefiting patient well-being and reducing the current burden on diagnostic services. Our team has initiated a prospective, observational cohort study in East London (MOTION study - NCT07035691), enrolling 2WW patients referred to a tertiary care unit, to assess the diagnostic performance of hPG80 in a diverse, real-world symptomatic population.^60^ This is particularly relevant given the rising CRC burden in socioeconomically deprived and ethnically diverse urban communities, where factors such as access to care and awareness can significantly impact diagnostic timeliness.

### 4.3. hPG80 as an Adjunct to FIT and a Surrogate Blood Marker

The integration of hPG80, either as an adjunct to existing FIT-based tools or as a surrogate blood marker, holds significant promise for optimising CRC screening and diagnostic pathways. Preliminary evidence suggests that hPG80 may outperform FIT in certain settings, particularly in detecting proximal or non-bleeding lesions that might escape stool-based detection. Furthermore, a combined diagnostic approach integrating hPG80 with FIT or other blood-based markers may yield synergistic benefits, enhancing both sensitivity and specificity. Economically, the introduction of hPG80 could significantly reduce downstream costs by decreasing the volume of inappropriate or negative colonoscopies, thereby conserving endoscopy resources and shortening diagnostic delays. A cost-utility analysis considering quality-adjusted life years (QALYs), direct costs, and productivity losses would be essential to support widespread adoption.^61^ On current evidence, hPG80 is best positioned as an adjunctive triage marker rather than a stand-alone replacement for FIT or colonoscopy. Its elevation across multiple tumour types limits its specificity as a single primary screening test, so a realistic near-term role is to refine risk within a defined symptomatic or FIT-positive group, where the pre-test probability of colorectal cancer is already raised and a competing tumour source is less likely. A second plausible role is post-treatment surveillance, where serial within-patient change is interpreted rather than a single population threshold. In both roles, hPG80 would sit alongside, not against, the validated cell-free DNA platforms. Because hPG80 reports a secreted protein and circulating tumour DNA tests report tumour-derived nucleic acids, the two signals are biologically orthogonal, and a multi-marker panel that combines a protein analyte with a nucleic-acid analyte could in principle improve sensitivity for early or non-shedding lesions. These combinations remain hypothetical and require prospective head-to-head evaluation before any algorithmic position can be claimed.

### 4.4. Patient Acceptability and Patient-Reported Outcomes

The case for a blood-based marker rests partly on acceptability, not on accuracy alone. A test that patients are willing to take repeatedly may detect more disease at the population level than a more sensitive test with poor uptake. This is the rationale behind the observation that patients offered a blood test completed screening at higher rates than those offered stool testing or colonoscopy, and it is central to any claim that hPG80 could improve participation. If the review advocates integration into screening and symptomatic pathways, then acceptability, quality of life, and patient preference become primary considerations rather than secondary ones. Patient-reported outcomes provide the structured means to capture these dimensions, and they are increasingly recognised as essential to oncology evaluation and to regulatory and health-technology decision-making.^62^ We therefore recommend that prospective evaluation of hPG80 embed validated patient-reported outcome and acceptability measures alongside diagnostic endpoints, so that the test is judged on the patient experience of screening and not only on sensitivity and specificity. The MOTION study offers an opportunity to collect these measures in a diverse symptomatic population.

### 4.5. Stage of CRC

Emerging data support hPG80 as a dynamic biomarker of disease activity. Rising levels may precede radiographic progression or clinical relapse, making it potentially valuable in post-operative surveillance or in monitoring treatment response. The observed reduction in hPG80 following surgery in patients with metastatic gastrointestinal cancer towards the peritoneum further supports its dynamic nature in the context of disease progression and treatment response.^33^ These dynamic properties suggest a role for hPG80 not only in diagnosis but also in longitudinal disease monitoring and secondary prevention, enabling more effective risk stratification within adenoma surveillance programmes and ensuring healthcare resources are directed towards individuals at higher risk of recurrence.

### 4.6. Benign/Non-Cancerous Polyps

Some evidence suggests that rising hPG80 levels may be associated with the progression of CRC and could serve as a marker for liver metastasis as well.^63,64^ This indicates its potential to serve as a marker for high-grade dysplasia or advanced CAAs, enabling earlier intervention and potentially preventing malignant transformation. This could be particularly valuable in adenoma surveillance programs, allowing for a more targeted approach to follow-up. There is ongoing debate about the natural history of small benign polyps (less than 5 mm in size) and the threshold at which a polypectomy is warranted. Future research should explore the correlation between hpG80 levels and the natural biological progression of polyps.

### 4.7. Other Colorectal Pathologies

While the primary focus of this review is on the diagnosis of CRC, the potential of hPG80 to serve as a marker for liver metastasis has also been suggested.^63,64^ CRC can co-exist with other benign pathologies such as diverticular disease and inflammatory bowel disease (IBD). Future research should focus on specific patterns of hpG80 expression and its ability to differentiate cancer diagnosis in such clinical context.

### 4.8. Therapeutic Potential of hPG80

Preclinical findings highlight the therapeutic potential of targeting hPG80 with monoclonal antibodies, particularly in the subgroup of CRC patients with K-RAS mutations, for whom effective targeted treatment is currently lacking.^65^ Experimental studies suggest that monoclonal antibodies directed against hPG80 may inhibit tumour growth and metastasis.^33^ Given the observed lack of side effects in preclinical studies reported by Prieur et al, this approach offers a promising avenue for treating a significant proportion of CRC patients.^65^ Such therapies could complement existing regimens, offering a precision medicine approach for molecularly defined CRC subgroups.

### 4.9. Comparison With CEA for Treatment Response and Minimal Residual Disease Monitoring

Carcinoembryonic antigen (CEA) is currently the most commonly used serum biomarker in the post-treatment surveillance of CRC, particularly for monitoring response to chemotherapy and detecting recurrence. However, its clinical utility is limited by suboptimal sensitivity, especially in early-stage disease, and by the fact that a significant proportion of patients estimated at up to 30% being CEA non-producers. Moreover, CEA levels may be elevated in a variety of benign conditions, including smoking and inflammatory disorders, further reducing its specificity. In contrast, circulating hPG80 may represent a more dynamic and biologically relevant biomarker of disease activity. Several studies included in this review report a significant decline in hPG80 levels following curative tumour resection, suggesting a close association with tumour burden. Longitudinal data also indicate that hPG80 levels may correlate with treatment response, making it a potentially valuable tool for real-time monitoring during systemic therapy.

Although direct head-to-head comparisons between hPG80 and CEA have not yet been undertaken, the available evidence suggests that hPG80 may offer particular value in patients with low or normal CEA levels, where traditional monitoring strategies are less effective. Furthermore, the rapid clearance of hPG80 from circulation following tumour removal, and its apparent specificity to tumour activity, support its potential role in detecting minimal residual disease. This could be especially relevant in postoperative settings where early identification of residual disease could influence adjuvant therapy decisions. In addition, given the limitations and costs associated with circulating tumour DNA (ctDNA) testing, hPG80 may provide a more accessible alternative for serial monitoring in both high- and low-resource settings. Prospective comparative studies are needed to define the relative performance of hPG80 alongside CEA and ctDNA in these critical areas of post-treatment management.

### 4.10. Limitations

Despite the promising findings, the current body of research on hPG80 has inherent limitations that necessitate further investigation. A key limitation in many early biomarker studies, including those reviewed, is the absence of rigorous validation procedures, leading to concerns about overfitting and inflated estimates of diagnostic accuracy. Specifically, several studies failed to implement best practices such as internal validation, external validation in independent cohorts, and appropriate corrections for multiple hypothesis testing. These omissions risk producing results that are not reproducible or generalizable.

The low prevalence of CRC in the general population further complicates validation efforts. When incidence is low, dividing datasets into statistically powered training and validation sets becomes difficult without compromising model performance. This issue underscores the value of collaborative, multicenter consortia capable of aggregating sufficiently large and diverse datasets. In this regard, the study by You et al stands out for its multicohort validation and robust performance metrics, including sensitivity, specificity, and area under the receiver operating characteristic curve.^33^ Nonetheless, even these findings require replication through prospective studies in distinct populations and clinical contexts to confirm hPG80’s diagnostic robustness. It is crucial to acknowledge that elevated hPG80 levels have also been reported in various other common cancer types, including upper gastrointestinal, lung, neuro and breast malignancy. The widespread detection of hPG80 across these malignancies presents a challenge for its exclusive application for CRC. If hPG80 assays were to be implemented without careful consideration of other potential primary tumour sites, there is a risk of generating false positive results, leading to unnecessary follow up and patient anxiety. The specificity question is sharpened by the wider gastrin and progastrin biology in neuroendocrine disease, and by the parallel literature on a related but distinct peptide. Chauhan et al reported markedly raised hPG80 in neuroendocrine neoplasms, including small-cell lung cancer, which confirms that progastrin elevation is not specific to colorectal cancer.^37^ A separate precursor, progastrin-releasing peptide (ProGRP), has been characterised as a circulating marker of pulmonary and non-pulmonary neuroendocrine neoplasms, and although ProGRP is molecularly distinct from progastrin and hPG80, the two share the limitation that elevation reflects neuroendocrine and other tumour activity rather than a single organ of origin.^66^ Taken together, these observations indicate that hPG80 cannot discriminate the tissue of origin on its own, and they reinforce that any colorectal application must control for competing primary tumour sites.

Biological heterogeneity also poses a challenge. The molecular subtypes of CRC like microsatellite instability-high, CpG island methylator phenotype, and CIN may differentially influence hPG80 expression. Future research should explore whether hPG80 levels vary across these molecular landscapes, and whether such variation might inform subtype-specific screening or therapeutic strategies.

Also, further longitudinal studies are crucial to determine the correlation between hPG80 levels and disease progression, treatment response, and overall survival. Investigating the molecular mechanisms underlying hPG80 overexpression in cancer cells could reveal novel therapeutic targets.

Furthermore, the methods employed in detecting and measuring the hPG80 levels vary across the literature. This is most probably the cause of the reported discrepancies of hPG80 concentrations and cut-off values. Older studies report RIA technique used. While RIA can offer high sensitivity, they rely on radioactive labels posing a challenge with handling, disposal and shelf-life of reagents. A notable drawback of older RIA methods, particularly for related peptides like gastrin, has reported cross-reactivity with other substances, potentially leading to less specific measurements and higher background noise, especially at lower, physiological concentrations. Recent studies report on ELISA technique as the predominant method. This method offers several advantages including safer reagents, faster turnaround times, and suitability for high-throughput analysis. This methodological shift has a direct bearing on comparability. The radioimmunoassay studies and the enzyme-linked immunosorbent assay studies do not measure the same thing on the same scale, so their reported cut-offs are not interchangeable. The radioimmunoassay thresholds in Siddheshwar et al and Konturek et al, expressed in the tens to low hundreds of picomolar, cannot be mapped onto the single-digit picomolar values from the DxPG80 assay, and a clinician could not transfer one study’s cut-off to another assay without recalibration. The case-control design shared by all five studies further inflates apparent separation between groups, because cases and controls were sampled from different populations rather than from a single screening or symptomatic cohort. None of the studies reported a prospectively locked threshold validated in an independent colorectal cohort. For these reasons, the consistent direction of effect across studies should be read as a qualitative signal of association rather than as evidence of a transferable, validated diagnostic cut-off.

Finally, comparative studies against existing tests are necessary to establish hPG80’s relative efficacy and potential advantages in clinical practice. Regulatory approval and clinical implementation will depend on clear evidence of clinical benefit, cost-effectiveness, and impact on patient outcomes metrics that are currently insufficiently addressed in the existing literature.

## 5. Conclusion

This scoping review affirms the significant potential of hPG80 as a blood-based biomarker for the early detection, diagnosis, and management of CRC. The synthesis rests on five heterogeneous studies, none of which reported a colorectal cancer-specific measure of diagnostic accuracy, and the included designs do not allow a pooled estimate. The consistent elevation of progastrin and hPG80 in colorectal cancer is best regarded as a reproducible association that supports further study, rather than as established diagnostic performance. hPG80 is not specific to colorectal cancer, so any clinical role is likely to be adjunctive, within a defined symptomatic or screen-positive group, or in serial post-treatment monitoring, rather than as a stand-alone primary screening test. Realising any clinical value will require prospective validation with locked thresholds in independent cohorts, direct comparison against FIT, carcinoembryonic antigen, and validated cell-free DNA platforms, formal cost-effectiveness analysis, and the collection of patient-reported and acceptability outcomes. The MOTION study is designed to begin addressing these questions in a symptomatic referral pathway.

## Supplemental Material

Supplemental Material - Unlocking Colorectal Cancer Diagnostics With Circulating Progastrin (hPG80): A Scoping Review Supplemental Material for Unlocking Colorectal Cancer Diagnostics With Circulating Progastrin (hPG80): A Scoping Review by Valentin Butnari, Deepak Kumar Dinkar, Jayan Dewantha Jayasinghe, Manthan Patel, Nirooshun Rajendran Sherif Raouf, Madapura Marulasiddappa Pradeepa, Mohamed Adhnan Thaha in Biomarker Insights.

## Data Availability

The data that supports the findings of this study are available upon reasonable requests from the authors.

## Appendix

### Abbreviations

CRC: Colorectal cancer
UK: United Kingdom
CAAs: Colorectal adenomas
FIT: Faecal immunochemical test
NHS: National Health Service
BCSP: Bowel Cancer Screening Programme
RIA: Radioimmunoassay
ELISA: Enzyme-linked immunosorbent assay
ROC: Receiver operating characteristic
CIN: Chromosomal instability pathway
QALYs: Quality-adjusted life years
hPG80: Human circulating progastrin (80 amino acid)
QUADAS-2: Quality Assessment of Diagnostic Accuracy Studies 2
NOS: Newcastle-Ottawa Scale
ctDNA: Circulating tumour DNA
CEA: Carcinoembryonic antigen
PRO: Patient-reported outcome
ProGRP: Progastrin-releasing peptide
eoCRC: Early-onset colorectal cancer

## References

[1] Sung H Ferlay J Siegel RL , et al. Global Cancer Statistics 2020: GLOBOCAN estimates of incidence and mortality worldwide for 36 cancers in 185 countries. CA a Cancer Journal for Clinicians. 2021;71(3):209-249. doi:10.3322/caac.21660. 33538338

[2] Ferlay J Colombet M Soerjomataram I , et al. Cancer statistics for the year 2020: An overview. International Journal of Cancer. 2021;149(4):778-789. doi:10.1002/ijc.33588. 33818764

[3] Cancer Research UK . Bowel cancer statistics. Cancer Research UK; 2025. Available from: https://www.cancerresearchuk.org/health-professi.onal/cancer-statistics/statistics-by-cancer-type/bowel-cancer

[4] Morgan E Arnold M Gini A , et al. Global burden of colorectal cancer in 2020 and 2040: incidence and mortality estimates from GLOBOCAN. Gut. 2023;72:338-344. doi:10.1136/gutjnl-2022-327736. 36604116

[5] Luo Q Smith DP . Global cancer burden: progress, projections, and challenges. The Lancet. 2025;406(10512):1536-1537. doi:10.1016/S0140-6736(25)01570-3. 41015053

[6] Sung H Siegel RL Laversanne M , et al. Colorectal cancer incidence trends in younger versus older adults: an analysis of population-based cancer registry data. The Lancet Oncology. 2025;26(1):51-63. doi:10.1016/S1470-2045(24)00600-4. PMC11695264 39674189

[7] Balchen V Simon K . Colorectal cancer development and advances in screening. Clinical Interventions in Aging. 2016;11:967-976. doi:10.2147/cia.s109285. PMC4958365 27486317

[8] Sawicki T Ruszkowska M Danielewicz A Niedźwiedzka E Arłukowicz T Przybyłowicz KE . A review of colorectal cancer in terms of epidemiology, risk factors, development, symptoms and diagnosis. Cancers. 2021;13(9):2025. doi:10.3390/cancers13092025. PMC8122718 33922197

[9] Nguyen LH Goel A Chung DC . Pathways of colorectal carcinogenesis. Gastroenterology. 2019;158(2):291-302. doi:10.1053/j.gastro.2019.08.059. PMC6981255 31622622

[10] Muller AD Sonnenberg A . Prevention of colorectal cancer by flexible endoscopy and polypectomy: A Case-Control Study of 32 702 veterans. Annals of Internal Medicine. 1995;123(12):904-910. doi:10.7326/0003-4819-123-12-199512150-00002. 7486484

[11] Zauber AG Winawer SJ O’Brien MJ , et al. Colonoscopic polypectomy and Long-Term prevention of Colorectal-Cancer deaths. New England Journal of Medicine. 2012;366(8):687-696. doi:10.1056/nejmoa1100370. PMC3322371 22356322

[12] World Health Organization: WHO . Cancer - Screening and early detection. 2010. Available from: https://www.who.int/europe/news-room/fact-sheets/item/cancer-screening-and-early-detection-of-cancer

[13] Liu SC Zhang H . Early diagnostic strategies for colorectal cancer. World Journal of Gastroenterology. 2024;30(33):3818-3822. doi:10.3748/wjg.v30.i33.3818. PMC11438623 39351429

[14] Lopes SR Martins C Santos IC Teixeira M Gamito É Alves AL . Colorectal cancer screening: A review of current knowledge and progress in research. World Journal of Gastrointestinal Oncology. 2024;16(4):1119-1133. doi:10.4251/wjgo.v16.i4.1119. PMC11037045 38660635

[15] Shaukat A Levin TR . Current and future colorectal cancer screening strategies. Nature Reviews Gastroenterology & Hepatology. 2022;19(8):521-531. doi:10.1038/s41575-022-00612-y. PMC9063618 35505243

[16] Cusumano VT May FP . Making FIT count: Maximizing appropriate use of the fecal immunochemical test for colorectal cancer screening programs. Journal of General Internal Medicine. 2020;35(6):1870-1874. doi:10.1007/s11606-020-05728-y. PMC7280423 32128688

[17] Maxwell S Weller D Dennison B , et al. Stratified/risk-based screening for colorectal cancer in the UK: an overview. Colorectal Cancer. 2025;14(1):2501851. doi:10.1080/1758194x.2025.2501851. PMC12309461 40747266

[18] Monahan KJ Davies MM Abulafi M , et al. Faecal immunochemical testing (FIT) in patients with signs or symptoms of suspected colorectal cancer (CRC): a joint guideline from the Association of Coloproctology of Great Britain and Ireland (ACPGBI) and the British Society of Gastroenterology (BSG). Gut. 2022;71(10):1939-1962. doi:10.1136/gutjnl-2022-327985. PMC9484376 35820780

[19] Chuang MPC Chiu HM . Does colonoscopy as a first screening test still make Sense?- Counterpoint. Digestive Diseases and Sciences. 2024;70(5):1606-1615. doi:10.1007/s10620-024-08695-0. 39641898

[20] Bowel cancer screening standards data report 2023-24. GOV.UK. 2025. Available from: https://www.gov.uk/government/publications/bowel-cancer-screenin.g-annual-report-2023-to-2024/bowel-cancer-screening-standards-data-report-2023-24

[21] Wender RC Doroshenk M Brooks D Hotz J Smith RA . Creating and implementing a national public health campaign: the American Cancer Society’s and National Colorectal Cancer Roundtable’s 80% by 2018 initiative. The American Journal of Gastroenterology. 2018;113(12):1739-1741. doi:10.1038/s41395-018-0384-1. PMC6768590 30413821

[22] Coronado GD Jenkins CL Shuster E , et al. Blood-based colorectal cancer screening in an integrated health system: a randomised trial of patient adherence. Gut. 2024;73(4):gutjnl-2023. doi:10.1136/gutjnl-2023-330980. 38176899

[23] Shweikeh F Zeng Y Jabir AR , et al. The emerging role of blood-based biomarkers in early detection of colorectal cancer: A systematic review. Cancer Treatment and Research Communications. 2025;42:100872. doi:10.1016/j.ctarc.2025.100872. 39892077

[24] Mannucci A Goel A . Stool and blood biomarkers for colorectal cancer management: an update on screening and disease monitoring. Molecular Cancer. 2024;23(1):259. doi:10.1186/s12943-024-02174-w. PMC11575410 39558327

[25] Chatterjee N Shi J García-Closas M . Developing and evaluating polygenic risk prediction models for stratified disease prevention. Nature Reviews Genetics. 2016;17(7):392-406. doi:10.1038/nrg.2016.27. PMC6021129 27140283

[26] Dunlop MG Tenesa A Farrington SM , et al. Cumulative impact of common genetic variants and other risk factors on colorectal cancer risk in 42 103 individuals. Gut. 2012;62(6):871-881. doi:10.1136/gutjnl-2011-300537. PMC5105590 22490517

[27] Zygulska AL Pierzchalski P . Novel diagnostic biomarkers in colorectal cancer. International Journal of Molecular Sciences. 2022;23(2):852. doi:10.3390/ijms23020852. PMC8776048 35055034

[28] Liang PS Zaman A Kaminsky A , et al. Blood test increases colorectal cancer screening in persons who declined colonoscopy and fecal immunochemical test: a randomized controlled trial. Clinical Gastroenterology and Hepatology. 2023;21(11):2951-2957.e2. doi:10.1016/j.cgh.2023.03.036. PMC10523873 37037262

[29] Cavestro GM Mannucci A Balaguer F , et al. Delphi Initiative for Early-Onset Colorectal Cancer (DIRECt) International Management Guidelines. Clin Gastroenterol Hepatol. 2023;21(3):581-603. doi:10.1016/j.cgh.2022.12.006. PMC11207185 36549470

[30] Chung DC Gray DM Singh H , et al. A cell-free DNA blood-based test for colorectal cancer screening. N Engl J Med. 2024;390(11):973-983. doi:10.1056/NEJMoa2304714. 38477985

[31] Shaukat A Burke CA Chan AT , et al. Clinical validation of a circulating tumor DNA-based blood test to screen for colorectal cancer. JAMA. 2025;334(1):56-63. doi:10.1001/jama.2025.7515. PMC12131177 40455622

[32] Fioretzaki R Sarantis P Charalampakis N , et al. Progastrin: An overview of its crucial role in the tumorigenesis of gastrointestinal cancers. Biomedicines. 2024;12(4):885. doi:10.3390/biomedicines12040885. PMC11047876 38672239

[33] You B Mercier F Assenat E , et al. The oncogenic and druggable hPG80 (Progastrin) is overexpressed in multiple cancers and detected in the blood of patients. EBioMedicine. 2019;51:102574. doi:10.1016/j.ebiom.2019.11.035. PMC6938867 31877416

[34] Bardram L . Progastrin in serum from Zollinger-Ellison patients. Gastroenterology. 1990;98(6):1420-1426. doi:10.1016/0016-5085(90)91071-d. 2338186

[35] Siddheshwar RK Gray JC Kelly SB . Plasma levels of progastrin but not amidated gastrin or glycine extended gastrin are elevated in patients with colorectal carcinoma. Gut. 2001;48(1):47-52. doi:10.1136/gut.48.1.47. PMC1728168 11115822

[36] Konturek PC Bielanski W Konturek SJ , et al. Progastrin and cyclooxygenase-2 in colorectal cancer. Digestive Diseases and Sciences. 2002;47(9):1984-1991. doi:10.1023/a:1019652224424. 12353842

[37] Chauhan A Prieur A Kolesar J , et al. HPG80 (Circulating progastrin), a novel Blood-Based biomarker for detection of poorly differentiated neuroendocrine carcinoma and well differentiated neuroendocrine tumors. Cancers. 2022;14(4):863. doi:10.3390/cancers14040863. PMC8870162 35205614

[38] Paterson AC Macrae FA Pizzey C Baldwin GS Shulkes A . Circulating gastrin concentrations in patients at increased risk of developing colorectal carcinoma. Journal of Gastroenterology and Hepatology. 2013;29(3):480-486. doi:10.1111/jgh.12417. 24716212

[39] Hollande F Lee DJ Choquet A Roche S Baldwin GS . Adherens junctions and tight junctions are regulated via different pathways by progastrin in epithelial cells. Journal of Cell Science. 2003;116(7):1187-1197. doi:10.1242/jcs.00321. 12615962

[40] Singh P Owlia A Varro A Dai B Rajaraman S Wood T . Gastrin gene expression is required for the proliferation and tumorigenicity of human colon cancer cells. Cancer Research. 1996;56(18):4111-4115. 8797575

[41] Wu H Owlia A Singh P . Precursor peptide progastrin1-80reduces apoptosis of intestinal epithelial cells and upregulates cytochromecoxidase Vb levels and synthesis of ATP. American Journal of Physiology-Gastrointestinal and Liver Physiology. 2003;285(6):G1097-G1110. doi:10.1152/ajpgi.00216.2003. 12881229

[42] Giraud J Failla LM Pascussi JM , et al. Autocrine secretion of progastrin promotes the survival and Self-Renewal of colon cancer stem-like cells. Cancer Research. 2016;76(12):3618-3628. doi:10.1158/0008-5472.can-15-1497. 27197176

[43] Najib S Kowalski-Chauvel A Do C Roche S Cohen-Jonathan-Moyal E Seva C . Progastrin a new pro-angiogenic factor in colorectal cancer. Oncogene. 2014;34(24):3120-3130. doi:10.1038/onc.2014.255. 25109333

[44] Tricco AC Lillie E Zarin W , et al. PRISMA Extension for Scoping Reviews (PRISMA-SCR): Checklist and explanation. Annals of Internal Medicine. 2018;169(7):467-473. doi:10.7326/m18-0850. 30178033

[45] The Newcastle-Ottawa Scale (NOS) . Copyright 2011 Ottawa Hospital Research Institute. All Rights Reserved. Available from: https://www.ohri.ca/programs/clinical_epidemiology/oxford.asp

[46] Rehfeld JF Zhu X Norrbom C , et al. Prohormone convertases 1/3 and 2 together orchestrate the site-specific cleavages of progastrin to release gastrin-34 and gastrin-17. Biochemical Journal. 2008;415(1):35-43. doi:10.1042/bj20080881. 18554181

[47] Duan S Rico K Merchant JL . Gastrin: From physiology to gastrointestinal malignancies. Function. 2021;3(1):zqab062. doi:10.1093/function/zqab062. PMC8788842 35330921

[48] MacNicol JL Pearson W . Gastrin and nitric oxide production in cultured gastric antral mucosa are altered in response to a gastric digest of a dietary supplement. Frontiers in Veterinary Science. 2021;8:684203. doi:10.3389/fvets.2021.684203. PMC8520902 34671658

[49] Koh TJ Bulitta CJ Fleming JV Dockray GJ Varro A Wang TC . Gastrin is a target of the β-catenin/TCF-4 growth-signaling pathway in a model of intestinal polyposis. Journal of Clinical Investigation. 2000;106(4):533-539. doi:10.1172/jci9476. PMC380254 10953028

[50] Singh P Xu Z Dai B Rajaraman S Rubin N Dhruva B . Incomplete processing of progastrin expressed by human colon cancer cells: role of noncarboxyamidated gastrins. American Journal of Physiology-Gastrointestinal and Liver Physiology. 1994;266(3):G459-G468. doi:10.1152/ajpgi.1994.266.3.g459. 8166285

[51] Ciccotosto GD McLeish A Hardy KJ Shulkes A . Expression, processing, and secretion of gastrin in patients with colorectal carcinoma. Gastroenterology. 1995;109(4):1142-1153. doi:10.1016/0016-5085(95)90572-3. 7557079

[52] Kohli M Tan W Vire B , et al. Prognostic value of plasma HPG80 (Circulating progastrin) in metastatic renal cell carcinoma. Cancers. 2021;13(3):375. doi:10.3390/cancers13030375. PMC7864155 33498444

[53] Dupuy M Iltache S Rivière B , et al. Plasma HPG80 (Circulating progastrin) as a novel prognostic biomarker for hepatocellular carcinoma. Cancers. 2022;14(2):402. doi:10.3390/cancers14020402. PMC8774261 35053564

[54] Fearon ER Vogelstein B . A genetic model for colorectal tumorigenesis. Cell. 1990;61(5):759-767. doi:10.1016/0092-8674(90)90186-i. 2188735

[55] Shang S Hua F Hu ZW . The regulation of β-catenin activity and function in cancer: therapeutic opportunities. Oncotarget. 2017;8(20):33972-33989. doi:10.18632/oncotarget.15687. PMC5464927 28430641

[56] National Health Service Website . Bowel cancer screening. nhs.uk; 2025. Available from: https://www.nhs.uk/conditions/bowel-cancer-screening/

[57] Koo S Neilson LJ Von Wagner C Rees CJ . The NHS Bowel Cancer Screening Program: current perspectives on strategies for improvement. Risk Management and Healthcare Policy. 2017;10:177-187. doi:10.2147/rmhp.s109116. PMC5720037 29270036

[58] Tepus M Yau TO . Non-Invasive Colorectal Cancer Screening: An Overview. Gastrointestinal Tumors. 2020;7(3):62-73. doi:10.1159/000507701. PMC7445682 32903904

[59] Bailey JA Morton AJ Jones J , et al. Sociodemographic variations in the uptake of faecal immunochemical tests in primary care: a retrospective study. British Journal of General Practice. 2023;73(736):e843-e849. doi:10.3399/bjgp.2023.0033. PMC10587902 37845084

[60] ClinicalTrials.gov. Available from: https://www.clinicaltrials.gov/study/NCT07035691

[61] Ogden J . QALYs and their role in the NICE decision‐making process. Prescriber. 2017;28(4):41-43. doi:10.1002/psb.1562.

[62] Bellino S La Salvia A . The importance of patient reported outcomes in oncology clinical trials and clinical practice to inform regulatory and healthcare decision-making. Drugs R D. 2024;24(2):123-127. doi:10.1007/s40268-024-00478-2. PMC11315859 39023849

[63] Do C Bertrand C Palasse J Delisle MB Cohen-Jonathan-Moyal E Seva C . Activation of pro-oncogenic pathways in colorectal hyperplastic polyps. BMC Cancer. 2013;13(1):531. doi:10.1186/1471-2407-13-531. PMC3829387 24209454

[64] Westwood DA Patel O Christophi C Shulkes A Baldwin GS . Progastrin: a potential predictive marker of liver metastasis in colorectal cancer. International Journal of Colorectal Disease. 2017;32(7):1061-1064. doi:10.1007/s00384-017-2822-8. 28432443

[65] Prieur A Cappellini M Habif G , et al. Targeting the Wnt Pathway and Cancer Stem Cells with Anti-progastrin Humanized Antibodies as a Potential Treatment for K-RAS-Mutated Colorectal Cancer. Clinical Cancer Research. 2017;23(17):5267-5280. doi:10.1158/1078-0432.ccr-17-0533. 28600477

[66] La Salvia A Fanciulli G . Progastrin-releasing peptide as a diagnostic biomarker of pulmonary and non-pulmonary neuroendocrine neoplasms. Endocr Res. 2024;49(4):243-250. doi:10.1080/07435800.2024.2365895. 39041209

