# Supplementary material for: Unlocking Colorectal Cancer Diagnostics With Circulating Progastrin (hPG80): A Scoping Review
Source: Biomark Insights. 2026 Sep 25;21:11772719261478092. doi: 10.1177/11772719261478092 (PMC13768663; doi:10.1177/11772719261478092)
Supplement: Supplementary file 1 [file sj-pdf-1-bmi-10.1177_11772719261478092.pdf]

Supplementary Materials

QUADAS-2 rates risk of bias across four domains and applicability concerns across the first three domains. The reference standard applicability domain is shown for completeness.

Study	Patient selection	Index test	Reference standard	Flow and timing	Applic. patient selection	Applic. index test	Applic. reference standard
Siddheshwar et al. 2001	HIGH	UNCLEAR	LOW	UNCLEAR	HIGH	UNCLEAR	LOW
Konturek et al. 2002	HIGH	HIGH	LOW	HIGH	HIGH	UNCLEAR	LOW
Paterson et al. 2014	HIGH	HIGH	UNCLEAR	UNCLEAR	HIGH	UNCLEAR	LOW
You et al. 2020	HIGH	UNCLEAR	LOW	UNCLEAR	HIGH	LOW	LOW
Chauhan et al. 2022	HIGH	UNCLEAR	LOW	UNCLEAR	HIGH	LOW	LOW

Supplementary Appendix 1. Full electronic search strategy

Databases searched: Ovid MEDLINE, PubMed, Ovid EMBASE, and the Cochrane Library. Date range: 1 January 1990 to 31 January 2025. No language or study-design filters were applied at the search stage; design-based exclusions were applied during screening. Reference lists of included and review articles were hand-searched. The strategy combined a population concept with an index-test concept using Boolean operators.

Concept 1 (population):

("colorectal cancer" OR "colorectal neoplasm" OR "colorectal carcinoma" OR "colorectal adenoma" OR "colorectal polyp" OR "bowel cancer")

Concept 2 (index test):

("progastrin" OR "hPG80" OR "circulating progastrin" OR "gastrin precursor" OR "DxPG80")

Combined: Concept 1 AND Concept 2.

Records identified: 55. After duplicate removal and title, abstract, and full-text screening in duplicate, five studies met the inclusion criteria. The PRISMA flow of records is shown in the main-text figure. The exact field codes and per-database record counts will be supplied by the authors on request and should be inserted here at proof stage to match the registered protocol (PROSPERO CRD420250654595).
